# Rectal Lipoma in an Elderly Male: A Case Report

**DOI:** 10.7759/cureus.8366

**Published:** 2020-05-30

**Authors:** Dimitrios Anyfantakis, Paraskevi Karona, Miltiades Kastanakis

**Affiliations:** 1 Primary Care, Primary Health Care Centre of Kissamos, Chania, GRC; 2 First Department of Surgery, Saint George General Hospitla of Chania, Chania, GRC; 3 First Department of Surgery, Saint George General Hospital of Chania, Chania, GRC

**Keywords:** lipoma, rectal, management, transanal excision

## Abstract

Lipomas of the gastrointestinal tract are uncommon, benign non-epithelial tumors detected incidentally during surgery or endoscopy. Rectal lipomas are extremely rare. Patients may be asymptomatic or present with rectal bleeding, constipation, tenesmus and signs of intestinal obstruction. Preoperative diagnosis is challenging. Management consists of simple observation, endoscopic or laparoscopic removal, and open surgery.

We present a case of an elderly male admitted to the Department of Surgery of a general hospital in Crete, complaining of a protruding rectal mass during defecation. CT raised the diagnostic suspicion. The mass was removed by trans-anal excision. Histopathology of the resected specimen confirmed the diagnosis. The patient had an uneventful postoperative course and was discharged home at the second postoperative day.

## Introduction

Colonic lipomas represent unusual benign non-epithelial tumors detected incidentally during surgery or colonoscopy [1]. The entity was first described by Bauer in 1757 [1]. Ascending colon is the most frequent site that lipomas are found [1]. Here we report on an unusual case of rectal lipoma. A literature review on its current diagnosis and management is also performed.

## Case presentation

**Figure 1:**
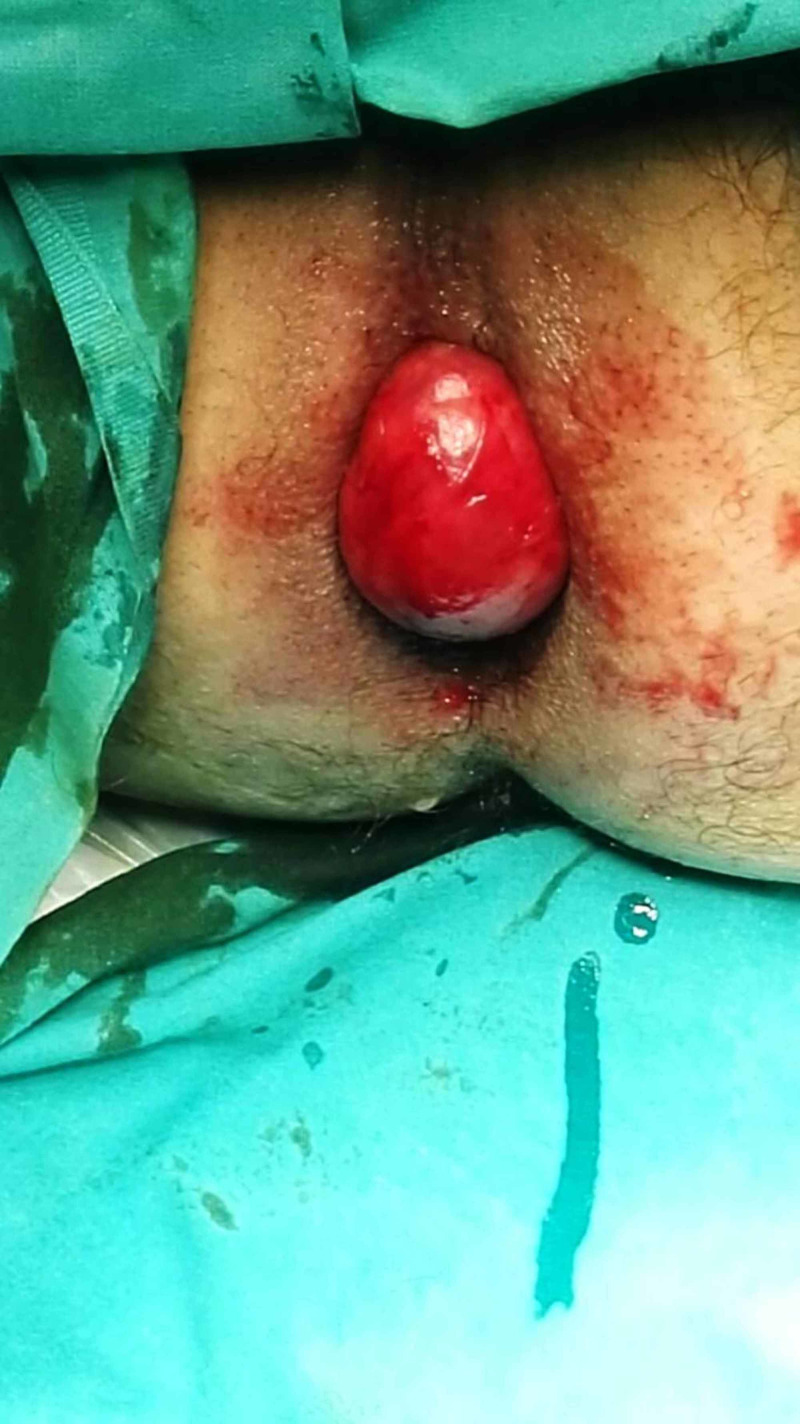
A hyperemic mass located in the anterior wall of the rectum

A 76-year-old man presented to the Surgery Department of the Saint George General Hospital of Chania, Crete, due to a rectal mass that protruded during defecation for the last four months. He denied a change in his bowel habits. The patient reported no history of anemia, episodes of diarrhea or rectal bleeding. Clinical examination was normal and no signs of systemic infection were noted. Rectal examination revealed a firm well-circumscribed hyperemic mass located in the anterior wall of the rectum, above the dentate line (Figure 1).

Results of the laboratory work-up including complete blood count, renal and liver function tests, blood coagulation and carcino-embryonic antigen were within normal limits. A colonoscopy was performed and a sub-mucosal mass was detected. Samples were obtained for histological examination, which was inconclusive.

**Figure 2:**
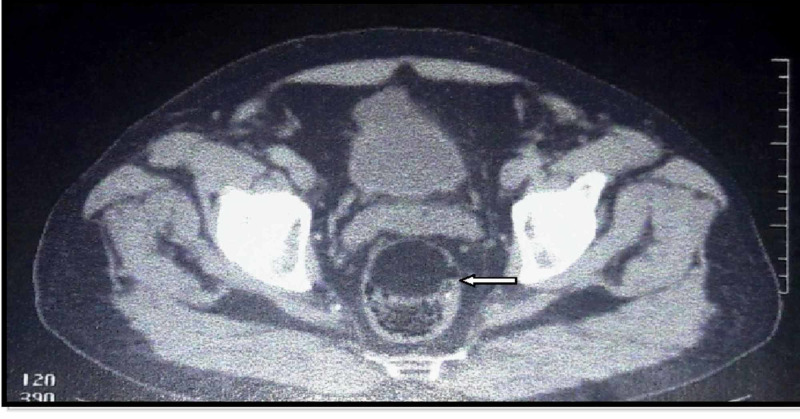
Contrast-enhanced abdominal CT showing a well-defined mass of 4.5 cm × 4.2 cm (arrow)

Contrast-enhanced abdominal CT disclosed the presence of a well-defined mass of 4.5 cm × 4.2 cm (Figure 2).

The patient was treated with trans-anal excision and the mass was removed with surgical enucleation. Biopsy of the specimen confirmed the diagnosis of rectal lipoma.

He had an uncomplicated clinical course and was discharged on the second postoperative day.

## Discussion

Colonic lipomas are rare, well-differentiated benign lesions and mostly arise from the sub-mucosa [2,3]. In 10% of cases, they extend to subserosa [2,3]. A frequency of 0.035%-4.4% has been reported worldwide with preponderance for female gender between the fifth and sixth decade of life [2,4].

Ιleo-cecal valve and cecum are most commonly involved, followed by the rectum, sigmoid and descending colon [5,6]. In an 18 years analysis among 17 patients with colonic lipomas, the rectal location was reported in only three patients [6]. Histopathological examination of lipomas reveals adipose tissue, which is surrounded by a fibrotic capsule [6]. Colonic lipomas are often asymptomatic [2,7]. However, if their size exceeds 2 cm, they may cause abdominal pain, rectal bleeding, alterations in bowel habits and intussusception [2]. Sometimes they may be presented as intestinal obstruction leading to inappropriate preoperative diagnosis [5].

The rarity of the disorder makes diagnosis challenging [2,3]. The differential diagnosis includes neoplastic lesions, atypical internal hemorrhoids with thrombosis, and rectal procidentia [2,3]. Barium enema radiography is a sensitive test and reveals a radiolucent lesion [2]. However, it lacks specificity and the patient may be misdiagnosed with a malignant lesion [2]. Ultrasound endoscopy is a helpful investigation in order to assess the extent of penetration into the muscularis propria [2]. Endoscopic examination of the colon represents a useful tool for the detection of lipomas, although it is less sensitive in ambiguous cases with atypical morphology [2,3]. Classical clinical signs during colonoscopy are the “tent sign” (lifting the overlying mucosa of the lipoma with forceps to create a tent-like shape), the “cushion sign”, (forceps shape a cavity of the lipoma which is resolved with their removal) and finally the “naked fat sign” described by Messer and Waye in 1982 (adipose tissue extracted from the lipoma during a biopsy) [2,8]. Histological sampling during colonoscopy for biopsy often fails to obtain adequate adipose lesion and the results remain inconclusive [2].

CT scan has been traditionally considered the most valuable imaging tool, especially when lipomas are large (>2 cm) [2,3,9]. Smaller lesions may be missed [2,3]. Preoperative investigations include full blood count, renal and liver function tests, and evaluation of cancer markers [4].

Management consists of observation for asymptomatic patients, endoscopic or surgical removal for symptomatic or those with associated complications [2,10]. Endoscopic intervention is considered a safe approach for pedunculated lipomas with a diameter of <2 cm [4]. Open surgery carries a lower complication risk compared with laparoscopic techniques [11]. For rectal lipomas located in the mid and upper rectum, trans-anal excision is the most favorable and feasible assessment.

## Conclusions

In this case, we aimed to highlight a rare presentation of an intestinal lipoma as a rectal prolapse. Accurate diagnosis of anal tumors poses a significant preoperative diagnostic difficulty. Surgeons and clinicians involved have to be aware of this rare entity and consider this in the differential diagnosis of patients admitted with rectal prolapse.
